# Olfactory function and viral recovery in COVID‐19

**DOI:** 10.1002/brb3.2006

**Published:** 2021-01-19

**Authors:** Marco Mazzoli, Maria Angela Molinari, Manuela Tondelli, Giada Giovannini, Riccardo Ricceri, Ludovico Ciolli, Livio Picchetto, Stefano Meletti

**Affiliations:** ^1^ Neurology Unit OCB Hospital Azienda Ospedaliera‐Universitaria Modena Italy; ^2^ Department of Biomedical, Metabolic and Neural Science University of Modena and Reggio Emilia Modena Italy; ^3^ Azienda USL Modena Italy

**Keywords:** anosmia, COVID‐19, hyposmia, SARS‐CoV‐2, smell

## Abstract

**Background:**

Olfactory and taste disorders were reported in up to 30%–80% of COVID‐19 patients. The purpose of our study was to objectively assess smell impairment in COVID‐19 patients and to correlate olfactory function with viral recovery.

**Methods:**

Between 15 and 30 April 2020, hospitalized patients with confirmed SARS‐CoV‐2 infection underwent an objective assessment of olfactory function with the Smell Identification subtest of the Sniffin’ Sticks Test (SI‐SST). Association between viral recovery and SI‐SST performance was evaluated.

**Results:**

51 patients were enrolled (49% males, mean age 66.2 ± 14.6 years). At the time of test administration, 45% were clinically recovered and 39% were virus‐free. Objective hyposmia/anosmia was found in 45% of the patients. Subjective olfactory disorders showed no association with the clinical or viral recovery status of the patients. On the contrary, none of the patients with anosmia and the 5% of hyposmic patients at test had viral recovery. The relative risk for hyposmic patients to be still positive at swab test was 10.323 (95% CI 1.483–71.869, *p* < .0001). Logistic regression analysis showed an independent and significant correlation between viral clearance and SI‐SST scores (OR = 2.242; 95% CI 1.322–3.802, *p* < .003). ROC curve analysis confirmed that a SI‐SST > 10.5 predicts viral clearance with 79% sensitivity and 87% specificity (AUC = 0.883).

**Conclusion:**

Hyposmia is part of COVID‐19 symptoms; however, only objectively assessed olfactory function is associated with viral recovery. SI‐SST is an easy and safe instrument, and further large multicentric studies should assess its value to predict infection and recovery.

## Graphical abstract

We conducted a prospective cross‐sectional study in consecutive hospitalized patients with confirmed SARS‐CoV‐2 infection to assess smell impairment and determine whether olfactory deficit could be a marker of active infection. Patients were asked about subjective olfactory and taste disorders and underwent an objective assessment of olfactory function with the Smell Identification subtest of the Sniffin’ Sticks Test. Besides confirming that olfactory deficits are present in about half of the patients, study main finding showed that none of the patients with anosmia and the 5.8% of the patients with hyposmia showed a viral recovery. The relative risk for hyposmic patients to be still positive at swab test was 10.323 (95% CI 1.483–71.869, *p* < .0001). Our study objectively confirmed that hyposmia is part of COVID‐19 symptoms. The objective assessment of olfactory function has higher sensitivity and reliability than self‐reported measurements.

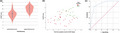

## 1 INTRODUCTION

During coronavirus disease 19 (COVID‐19) pandemic, several symptoms have been reported indicating a neurological involvement (Zubair et al., 2020), and among them, alteration of smell and taste perception are frequently observed (Beltrán‐Corbellini et al., 2020; Giacomelli et al., 2020; Lechien et al., 2020; Printza & Constantinidis, 2020). The presence of olfactory and taste disorders (OTDs) was reported in up to 30%–80% of COVID‐19 patients across different studies (Beltrán‐Corbellini et al., 2020; Giacomelli et al., 2020; Lechien et al., 2020; Printza & Constantinidis, 2020). However, these studies are based on subjective measurements, such as self‐reported questionnaires, without assessing the presence of hyposmia with validated tests. Furthermore, there is no information about the time course of hyposmia and its correlation between clinical and viral recovery. The aim of our study was to objectively evaluate smell impairment in COVID‐19 patients and determine olfactory function with respect to viral recovery.

## 2 MATERIALS AND METHODS

### 2.1 Patients and definitions

All patients were prospectively recruited during their stay at the COVID Unit of the University Hospital of Modena, Italy, between 15 and 30 April 2020. All had COVID‐19 diagnosis confirmed by at least one positive real‐time reverse transcriptase–polymerase chain reaction (RT‐PCR) assay for SARS‐CoV‐2 on rhinopharyngeal swab, in association with typical clinical and instrumental picture. Patients with chronic nasal diseases or neurological conditions associated with anosmia were excluded. Clinical, demographic, laboratory findings and treatments were collected. Severity of respiratory function was assessed considering the necessity of oxygen implementation, noninvasive ventilation (NIV), or invasive mechanical ventilation (IMV) during hospitalization. As suggested by most of the international guidelines, patients were defined as clinically recovered if they remained oxygen‐free, with peripheral blood oxygen saturation (SpO_2_) >94% and respiratory rate < 22 apm and without fever for at least 72 hr. Viral recovery was defined as negative SARS‐CoV‐2 RT‐PCR assays on two consecutive rhinopharyngeal swabs at least 24 hr apart.

### 2.2 Procedures

Each patient was asked about the presence and characteristics of OTD during the disease with a specific form. Olfactory function was objectively assessed using the Smell Identification subtest of the Sniffin’ Sticks Test (SI‐SST) (Oleszkiewicz et al., 2019). The examiner was blinded for viral recovery status at test time. SI‐SST is composed of 16 pens, each one equipped with a swab soaked with a 4 ml quantity of a specific smell. Each pen was presented close to both nostrils for 3 seconds: subjects were asked to match the smell with one of four alternatives written on a paper sheet. We defined hyposmia as an adjusted score ≤ 10^th^ percentile according to literature normative values reported for each age and gender group classification (Oleszkiewicz et al., 2019); therefore, every patient was classified as hyposmic or not according to the specific age and gender group value; functional anosmia was defined as a score < 8 points (Oleszkiewicz et al., 2019).

### 2.3 Statistical analysis

Statistical analyses were performed with SPSS version 24.0. Comparison of clinical data and smell performance were performed using parametric or nonparametric statistic as appropriate. Logistic regression was performed in order to test the relationship between viral recovery and smell test performance. Smell test diagnostic performance was also assessed by areas under the curve (AUCs) with 95% confidence intervals obtained by receiver operating characteristic (ROC) curve.

### 2.4 Standard protocol approvals, registration, and patient consents

The Human Ethic Committee of the University of Modena and Reggio Emilia approved this study, and written informed consent was obtained from all patients. The study conforms with World Medical Association Declaration of Helsinki.

### 2.5 Data availability statement

Anonymized data that support the findings of this study are available from the corresponding author upon reasonable request.

## 3 RESULTS

**FIGURE 1:**
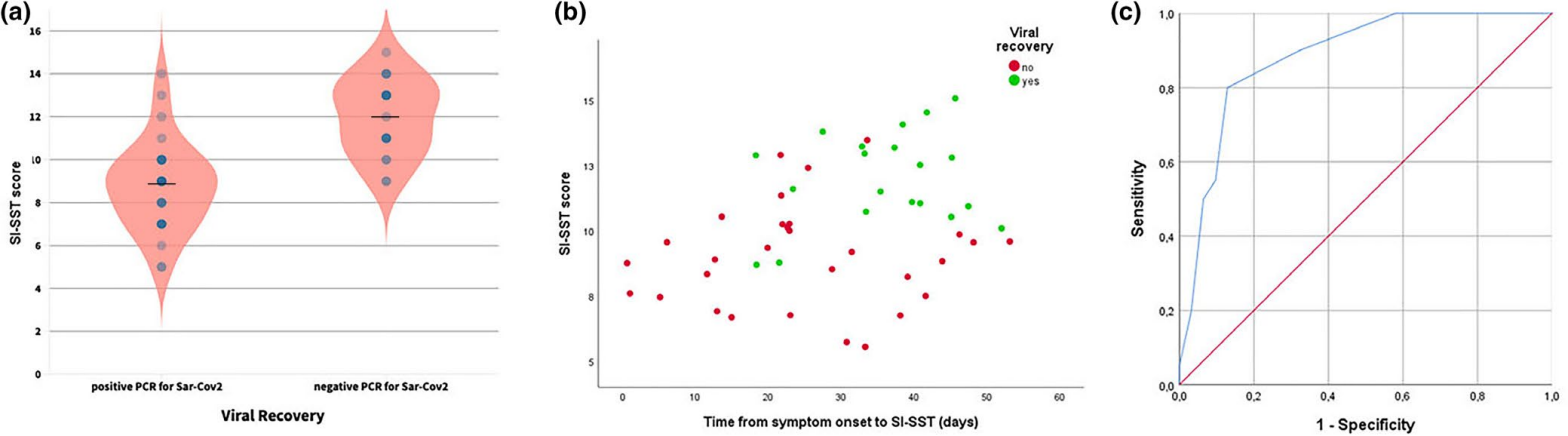
(a) Violin plots showing SI‐SST score distribution in patients with or without viral recovery (negative at 2 consecutive 24 hr apart nasopharyngeal swabs). The black line represents the mean score of each group. (b) Scatterplot showing SI‐SST score distribution as a function of time from symptom onset and viral recovery. (c) Receiver operating characteristic (ROC) curve for prediction of viral recovery based on SI‐SST score

Fifty‐one patients were enrolled (24 males; mean age 66.2 ± 14.6 years). SI‐SST was administered after a mean time of 29.3 days from onset of COVID‐19 symptoms. Table 1 shows the main clinical and demographic features of the patients. At the time of test administration, 23 patients (45%) were clinically recovered and 20 (39%) were virus‐free. 26% and 37% of patients reported subjective olfactory or taste deficits, respectively. At SI‐SST, 17 (33%) and 13 (25%) patients resulted hyposmic or anosmic, respectively. Considering both scores, ≤10^th^ percentile cutoff and row score < 8 points (functional anosmia), 23 (45%) subjects presented a defective performance at SI‐SST. No association between hyposmia/anosmia status at SI‐SST and any of the clinical, laboratory, or treatment variables was observed, including the clinical recovery status. In particular, SI‐SST scores were not significantly different among patients who had different severity of respiratory involvement during the disease. Moreover, subjective smell dysfunction showed no association with the SI‐SST deficit. On the contrary, none of the patients with anosmia and only 5% of the patients with hyposmia showed a viral clearance at test time (Table 2). Hyposmia status was associated with a risk ratio of 10.323 (95% CI 1.483–71.869; *p* < .0001) for being still positive at rhinopharyngeal swab test. Considering viral recovery, SARS‐CoV‐2‐negative patients performed better at SI‐SST than SARS‐CoV‐2‐positive patients (mean score ± *SD*: 12.0 ± 2.5 vs. 8.8 ± 2.5 points, respectively) (Figure 1a,b). Multivariable logistic regression analysis showed that a 1‐point increment in SI‐SST score corresponds to a 2.2‐fold higher odds ratio of being virus‐free in two consecutive swabs (OR = 2.242; 95% CI 1.322–3.802, *p* < .003), and this finding was independent from sex, age, severity of respiratory impairment, and time between recovery and test administration. ROC curve analysis showed that a SI‐SST score > 10.5 points predicted viral clearance with 79% sensitivity and 87% specificity, with AUCs of 0.883 (Figure 1c). None of the other considered variables was significantly related to viral clearance.

**TABLE 1:** Clinical and demographic characteristics (*N* = 51)

Gender, *n* (%)	
Male	25 (49%)
Female	26 (51%)
Age (years)	
Mean (±*SD*)	66.2 (±14.6)
Range	31–93
Time from onset to test (days)	
Mean (±*SD*)	29.3 (±13.3)
Range	1–54
Smokers, *n* (%)	16 (31%)
Subjective hyposmia, *n* (%)	13 (26%)
Subjective taste alteration, *n* (%)	19 (37%)
Symptoms, *n* (%)	
Fever	45 (88%)
Cough	29 (57%)
Dyspnea	37 (73%)
Diarrhea	4 (8%)
Headache	4 (8%)
Myalgia	8 (16%)
Diagnostics, *n* (%)	
Chest RX/CT scan	42 (82%)
Echo B‐lines	42 (82%)
Positive walking test	40 (78%)
Pulmonary thromboembolism	4 (8%)
Laboratory examinations, *n* (%)	
Leukopenia/lymphopenia	28 (55%)
High LDH	43 (84%)
High transaminases	17 (33%)
High gammaGT	6 (12%)
High CPK	9 (18%)
High D‐dimer	34 (67%)
Medical treatments, *n* (%)	
Hydroxychloroquine	44 (86%)
Azithromycin	42 (82%)
Steroids	28 (55%)
LMWH	49 (96%)
Tocilizumab	25 (49%)
Anakinra	3 (6%)
Remdesivir	3 (6%)
Respiratory support, *n* (%)	
None	10 (20%)
Low/high flow oxygen	13 (25%)
NIV	15 (30%)
Intubation and IMV	13 (25%)
Clinical recovery at test, *n* (%)^a^	23 (45%)
Time from clinical recovery to test (days)	
Mean (±*SD*)	7.7 (±7.3)
Range	1–28
Viral clearance at test, *n* (%)^b^	20 (39%)
Time from viral clearance to test (days)	
Mean (±*SD*)	7.0 (±5.9)
Range	1–22

Note

LDH, lactate dehydrogenase; GammaGT, gamma‐glutamyltransferase; CPK, creatine phosphokinase; LMWH, low‐molecular‐weight heparin; NIV, noninvasive ventilation; IMV, invasive mechanical ventilation.

^a^ Clinical recovery: Patients were considered clinically recovered if had no fever, respiratory rate < 22 apm, and peripheral blood oxygenation > 94% without oxygen implementation for the last 72 hr.

^b^ Viral clearance: Patients were considered virus‐free after two consecutive negative SARS‐CoV‐2 RT‐PCR assays on rhinopharyngeal swabs, separated by at least 24 hr.

**TABLE 2:** Supporting information: SI‐SST performance in relation to subjective hyposmia, clinical recovery and viral recovery (group comparison analysis)

SI‐SST performance	Overall sample (*N* = 51)	Subjective hyposmia			Clinical recovery			Viral recovery		
		Present (*N* = 13)	Absent (*N* = 38)	*p*	Present (*N* = 23)	Absent (*N* = 28)	*p*	Present (*N* = 20)	Absent (*N* = 31)	*p*
Mean score (±*SD*)	10.0 (±2.5)	10.8 (±2.5)	9.8 (±2.5)	.226	10.8 (±2.5)	9.4 (±2.5)	.059	12.0 (±2.5)	8.8 (±2.5)	**<.001**
Range	5–15	7–15	5–14		5–15	5–14		9–15	5–14	
Hyposmia, *n* (%)^a^	17 (33%)	4 (31%)	13 (34%)	.820	6 (26%)	11 (39%)	.320	1 (5%)	16 (52%)	**.001**
Functional anosmia, *n* (%)^b^	13 (26%)	3 (23%)	10 (26%)	.817	4 (17%)	9 (32%)	.229	0 (0%)	13 (42%)	**.001**
Combined hyposmia and anosmia, *n* (%)^c^	23 (45%)	5 (38%)	18 (47%)	.577	8 (35%)	15 (54%)	.180	1 (5%)	22 (71%)	**<.001**

Note

Groups were compared using chi‐square test for dichotomous variables. Significance: *p* < .05, two‐tailed (in bold).

^a^ Hyposmia: defined as SI‐SST score < 10^th^ percentile, adjusted for age and sex.

^b^ Functional anosmia: defined as SI‐SST score < 8 points.

^c^ Combined hyposmia and anosmia: defined as SI‐SST score < 10^th^ percentile, adjusted for age and sex, or SI‐SST raw score < 8 points.

## 4 DISCUSSION

We objectively assessed olfactory function by means of the SI‐SST, which is easy and rapid to perform at bedside (8–10 minutes) and it is provided with age‐ and gender‐adjusted normative values (Oleszkiewicz et al., 2019). In our cohort, subjective smell dysfunction was reported by 26% of the patients, while objectively assessed hyposmia/anosmia was present in 45% of patients. This means that self‐reported surveys of olfactory function may be unreliable in identifying hyposmic COVID‐19 patients. Indeed, previous studies already revealed that many hyposmic subjects may be unaware of hyposmia, particularly older individuals (Cavazzana et al., 2018; Landis et al., 2003). A recent meta‐analysis confirmed that the methodology used to assess olfactory function had a deep impact on smell performance prevalence rate identification: the pooled prevalence estimate of smell loss was 77% when assessed through objective measurements and 45% with subjective measurements, suggesting that subjective measures may underestimate the true prevalence of smell loss (Hannum et al., 2020). In line with this view, our results confirmed that objective methods are a more accurate method to identify smell loss as a result of infection with SARS‐CoV‐2. The most relevant finding of our study regards how hyposmia/anosmia correlates with viral healing, unlike subjective smell dysfunction report. However on a limited number of patients, no patients with functional anosmia and only 5% of hyposmic patients were negative at SARS‐CoV‐2 rhinopharyngeal swab. These data need to be confirmed on a larger population, but they suggest that SI‐SST could be very useful in screening patients for viral recovery, saving time and resources compared with rhinopharyngeal swab. In addition, objective evaluation of olfactory function may be helpful in screening patients or case contacts. It is relevant to us to underline that such test could provide real‐time information at low cost. A study limitation is that we only considered hospitalized patients: this may represent a selection bias, with prevalent inclusion of patients with more severe clinical manifestations. A larger sample size may be required to draw conclusive inferences to the whole COVID‐19 population. In addition, we are aware that other reasons than COVID‐19 may cause olfactory disfunction and that in general population, it is reported that up to 20% of the people are hyposmic and up to 5% are anosmic (Hummel et al., 2017). Pre‐COVID‐19 objective smell status was not available in our population. However, premorbid objective information about olfactory function in people infected by SARS‐CoV‐2 would be not easily available even in larger studies. Despite this point, we think that the relationship demonstrated between viral recovery and SI‐SST scores is reasonably specific for our COVID‐19 patients. Longitudinal studies will be useful to partially address this issue. Finally, further characterization of patients should be important. In particular, we did not acquire brain MRI of hyposmic patients. Recently, abnormal MRI signal involving the right gyrus rectus and the olfactory bulbs has been reported in a young COVID‐19 patient complaining of hyposmia and dysgeusia (Politi et al., 2019). In conclusion, our study confirmed that hyposmia is part of COVID‐19 symptoms. The objective assessment of olfactory function has higher sensitivity and reliability than self‐reported measurements. Moreover, smell function integrity is a predictor of viral clearance. SI‐SST is a cheap, easy, and safe instrument that can help in establishing COVID‐19 diagnosis and identifying virus‐free patients.

## CONFLICT OF INTEREST

M. Mazzoli, M.A. Molinari, M. Tondelli, G. Giovannini, R. Ricceri, L. Ciolli, and L. Picchetto report no disclosures. Stefano Meletti received research grant support from the Ministry of Health (MOH) and from the nonprofit organization foundation "Fondazione Cassa di Risparmio di Modena ‐ FCRM"; and has received personal compensation as scientific advisory board member for UCB and EISAI.

## AUTHOR CONTRIBUTIONS

Marco Mazzoli and Maria Angela Molinari designed and conceptualized the study, played a major role in the acquisition of data, analyzed and interpreted the data, and drafted the manuscript for intellectual content. Manuela Tondelli designed and conceptualized the study, analyzed and interpreted the data, and revised the manuscript for intellectual content. Giada Giovannini, Riccardo Ricceri, Ludovico Ciolli, and Livio Picchetto played a major role in the acquisition of data and interpreted the data. Stefano Meletti designed and conceptualized the study, interpreted the data, and revised the manuscript for intellectual content.

### Peer Review

The peer review history for this article is available at https://publons.com/publon/10.1002/brb3.2006.
